# Truncating Variant in *Myof* Gene Is Associated With Limb-Girdle Type Muscular Dystrophy and Cardiomyopathy

**DOI:** 10.3389/fgene.2019.00608

**Published:** 2019-06-26

**Authors:** Artem Kiselev, Raquel Vaz, Anastasia Knyazeva, Alexey Sergushichev, Renata Dmitrieva, Aleksandr Khudiakov, John Jorholt, Natalia Smolina, Ksenia Sukhareva, Yulia Fomicheva, Evgeny Mikhaylov, Lubov Mitrofanova, Alexander Predeus, Gunnar Sjoberg, Dmitriy Rudenko, Thomas Sejersen, Anna Lindstrand, Anna Kostareva

**Affiliations:** ^1^Department of Molecular Biology and Genetics, Almazov National Medical Research Centre, Saint Petersburg, Russia; ^2^Department of Molecular Medicine and Surgery, Centre for Molecular Medicine, Karolinska Institutet, Stockholm, Sweden; ^3^Computer Technologies Laboratory, ITMO University, Saint Petersburg, Russia; ^4^Department of Women’s and Children’s Health, Karolinska Institutet, Stockholm, Sweden; ^5^Arrhythmia Department, Almazov National Medical Research Centre, Saint Petersburg, Russia; ^6^Bioinformatics Institute, Saint Petersburg, Russia; ^7^Clinical Genetics, Karolinska University Laboratory, Karolinska University Hospital, Stockholm, Sweden

**Keywords:** myoferlin, haploinsufficiency, cardiomyopathy, muscular dystrophy, zebrafish

## Abstract

Even though genetic studies of individuals with neuromuscular diseases have uncovered the molecular background of many cardiac disorders such as cardiomyopathies and inherited arrhythmic syndromes, the genetic cause of a proportion of cardiomyopathies associated with neuromuscular phenotype still remains unknown. Here, we present an individual with a combination of cardiomyopathy and limb-girdle type muscular dystrophy where whole exome sequencing identified myoferlin (*MYOF*)—a member of the Ferlin protein family and close homolog of *DYSF*—as the most likely candidate gene. The disease-causative role of the identified variant c.[2576delG; 2575G>C], p.G859QfsTer8 is supported by functional studies *in vitro* using the primary patient’s skeletal muscle mesenchymal progenitor cells, including both RNA sequencing and morphological studies, as well as recapitulating the muscle phenotype *in vivo* in zebrafish. We provide the first evidence supporting a role of *MYOF* in human muscle disease.

## Introduction

Approximately 25% of genes associated with cardiomyopathy also cause neuromuscular disorders, and genetic studies of neuromuscular diseases have contributed substantially to uncover the molecular background of cardiac disorders (Bondue et al., 2018). The list of those genes was substantially extended due to availability of next-generation sequencing; however, the genetic cause of a proportion of cardiomyopathies associated with neuromuscular phenotype remains unknown.

Myoferlin is a member of the Ferlin protein family with a role in vesicle trafficking, membrane fusion, and repair (Posey et al., 2011). Two of these protein family members, Dysferlin (DYSF) and Otoferlin (OTOF), are well known in connection to human genetic disorders: *DYSF* gene was linked to limb-girdle muscle dystrophy 2B (LGMD2B) (MIM#253601) and Miyoshi myopathy (MIM#254130), while *OTOF* was reported as a causative gene for non-syndromic hearing loss (MIM#601071). Eventhough *DYSF* pathogenic variants are mainly associated with LGMD2B and Miyoshi myopathy, subtle cardiac dysfunction have also been described in patients with *DYSF* mutations (Wenzel et al., 2007; Choi et al., 2010; Nishikawa et al., 2016), and supported by a number of experimental studies (Han et al., 2007; Wang et al., 2015). These studies suggest a potential role for the Ferlin family of proteins in the development of cardiac and skeletal muscle disorders.

Myoferlin (*MYOF*) was first cloned in 2000 by Davis et al. and mainly described in connection to cancer cell invasion (Blomme et al., 2016; Zhang et al., 2018). In spite of extensive research, no phenotype linked to *MYOF* mutation has been reported yet (Davis et al., 2000; Bonne et. al., 2017). Nevertheless, *in vitro *studies showed that MYOF has a role in myoblast fusion, with loss of *MYOF* in the mouse model supporting this finding, as *MYOF*-null mice present with smaller myofibers due to reduced cell fusion (Doherty et al., 2005). Here, we present a clinical case of cardiomyopathy associated with limb-girdle type muscular dystrophy. Target sequencing of cardiomyopathy-associated genes did not identify any pathogenic variants. Utilizing whole exome sequencing (WES), we uncovered *MYOF* as the most likely candidate gene. Morphological and expression studies of the patient’s cells as well as a zebrafish knockdown model strongly support a role for *MYOF* variants in cardiac and skeletal muscle disorders.

## Materials and Methods

### Ethics Approval and Consent to Participate

The study was performed according to the Declaration of Helsinki, and approval was obtained from the Ethical Review Boards of Karolinska Institute and Almazov National Medical Research Centre, approval number 2016/54. Written informed consent was obtained from both the patient and healthy donors prior to the investigation, including a consent for publication. All procedures with zebrafish were performed in accordance with standard operating procedures approved by the Stockholm Ethical Board for Animal Experiments (permit number 13063-2017).

### Availability of Data and Materials

The dataset supporting the conclusions of this article is available in GEO repository (GSE119027) https://www.ncbi.nlm.nih.gov/geo/query/acc.cgi?acc=GSE119027 and can be found in the **Supplementary Material**.

### Genetic Testing

Target sequencing of 108 cardiomyopathy-associated genes (**Supplementary Material, Table S1**) and WES were performed as previously described (Kostareva et al., 2016; Kiselev et al., 2018) using a targeted panel of 108 cardiomyopathy-associated genes (**Supplementary Material, Table S1**). For *MYOF* gene, the NM_133337 reference sequence was used. After target and WES, variant classification was performed according to the guidelines from the American College of Medical Genetics (Richards et al., 2015).

### Skeletal Muscle Mesenchymal Progenitor Cell Purification and Separation

A muscle biopsy taken from the patient’s *m. deltoideus* was used for morphological examination and skeletal muscle mesenchymal progenitor cell (SM-MPC) isolation. Control cells were obtained from the hip muscles of healthy donors (n = 13). Skeletal muscle mesenchymal progenitor cells were isolated enzymatically, using an adaptation of published protocols (Danoviz and Yablonka-Reuveni, 2012; Smolina et al., 2015). In brief, muscle tissue was placed into enzyme solution, mechanically disrupted with scissors and digested for 60 min at 37 C in 5 ml filtered 0.1% collagenase I (C0130, Sigma, Germany). To remove collagenase and cell debris after digestion, the cell suspension was centrifuged for 5 min at 1,000×*g* and the supernatant containing enzyme solution was discarded. To release stem cells from the fibers, the pellet was resuspended using sterile pipette tips in 2.5 ml of washing media (DMEM supplemented with 10% horse serum; Gibco, USA). After the resuspension, the fibers were let to settle for 5 min and then the supernatant containing stem cells was replaced to a fresh tube. To increase yield, this step was repeated twice. The double-collected supernatant was filtered through a 40-μm nylon cell strainer and centrifuged for 10 min at 1,000 × *g*; the resultant supernatant was discarded and the pellet of cells was placed in proliferation media (DMEM supplemented with 10% FCS) on cell culture dishes and cultured until 80% confluence. Cell staining and Western blot analysis were performed using anti-myoferlin antibody (Abcam 76746). MYOF protein expression levels were quantified as relative densitometric units normalized to Ponceau S staining. Densitometry was performed in NIH ImageJ software (USA). Aggregates area after immunocytochemistry using anti-MYOF antibody was measured using ZEN software (Carl Zeiss, Germany). At least four fields for each condition were analyzed. Distribution of aggregate size was analyzed using R-Studio version 1.0.153 with R version 3.0.1. Statistical analysis was performed using GraphPad Prism version 5.00 for Windows (GraphPad Software, www.graphpad.com). For comparison of two groups, the Mann–Whitney test was used. P < 0.05 was considered statistically significant.

### Flow Cytometry Analysis

The myogenic nature of purified cells was evaluated by flow cytometry analysis performed on CytoFLEX (Beckman Coulter) as previously described (Awaya et al., 2012; Lapan and Gussoni, 2012); the following panel of antibodies was used to determine immunophenotype: anti-CD56 PC7 (Beckman Coulter, USA, A21692), anti-CD146 PE (Beckman Coulter, USA, A07483), anti-CD166 PE (Beckman Coulter, USA, A22361), anti-CD73 PE (BD Pharmingen, USA, 550257), anti-CD105 APC (R&D Systems, USA, FAB1097A-100), and anti-CD45 PC5 (Beckman Coulter, USA, A07785). Data were analyzed using the CytExpert 2.0 (Beckman Coulter). The phenotypic characteristics of the obtained cells are illustrated in the **Supplementary Material, Figure S2A**.

### Differentiation Protocols

Myogenic differentiation of SM-MPC was induced by replacement of proliferation media with differentiation media: DMEM supplemented with 2% of horse serum. Cell cultures were taken for further analysis on seventh day after induction when myotubes were clearly visualized (**Supplementary Material, Figure S2B**).

### RNA Isolation and Analysis

Total RNA was isolated with Trizol reagent (Sigma, USA) from SM-MPC as well as from the patient’s and the donor’s tissue biopsies. After isolation, RNA was quantified using Qubit 2.0 fluorometer (Life Technologies, Invitrogen division, Darmstadt, Germany) with Qubit RNA HS Assay Kit and Nanodrop 1000 spectrophotometer (Thermo Scientific, Wilmington, DE, USA). Additionally, total RNA was quantified using Bioanalyser 2100 (Agilent Technologies, Palo Alto, CA) RNA Nano kit; RINs for all samples were in the range >9. Libraries for RNA sequencing were prepared using TruSeq Stranded mRNA kit (Illumina, USA), according to the manufacturer’s recommendation. Single-read sequencing was performed on Illumina HiSeq 2500 instrument with TruSeq SR Cluster Kit v3 cBot, HS, and TruSeq SBS Kit v3 (50-cycles). Bcl files were processed to .fastq files using bcl2fastq Conversion Software v1.8.4 (Illumina). A quantitative evaluation of gene expression was performed with qPCR mix-HS SYBR+ROX (Evrogen, cat.no. PK156, Russia). Q-PCR data are presented as arbitrary units of mRNA expression normalized to GAPDH expression and to expression levels in reference sample. Sequences for Q-PCR primers can be found in **Supplementary Material, Table S2**.

RNA-seq data were quantified using RSEM with GENCODE annotation (Li and Dewey, 2011) and deposited in GEO repository (GSE119027). For pathways analysis, fgsea package was used (biorxiv, doi: https://doi.org/10.1101/060012) with MSigDB and Reactome collections; genes were ranked by log2-fold-change (Liberzon et al., 2015; Fabregat et al., 2018). The inflammation expression pattern was confirmed by comparison to GSE26852 dataset (Gene expression analysis of facioscapulohumeral muscular dystrophy muscle with different MRI pattern) (Tasca et al., 2012).

### Zebrafish Maintenance and Knockdown Studies

Adult zebrafish were maintained on a 14 h light/10 h dark cycle at the Karolinska Institute zebrafish core facility. Knockdown of endogenous *myof* was achieved by injecting 0.5-mM solution of a splice-modifying morpholino predicted to block intron 5 splicing and introduce a premature stop codon (5’ GCTGATCACACCGAAAAGTAAATGA 3’). Embryos expressing EGFP were positively selected at 24 hours post fertilization (hpf) and fixed at 48 hpf in 4% paraformaldehyde overnight at 4°C. Permeabilization was performed by incubation with 100% acetone for 20 min at −80°C, and immunolabelling was performed as previously described (Kiselev et al., 2018). Antibodies used were anti-GFP (ab290, AbCam), anti-Dystrophin and anti-Dystroglycan (7A10 and 7D11, D.S.H.B)., and anti-Rabbit Alexa488 and anti-Mouse Alexa594 (Life Technologies). Nuclear staining was performed by incubation with DAPI (4′,6-diamidino-2-phenylindole) at a final concentration of 10 µg/ml. Imaging was performed in 7–12 embryos per staining and experimental condition from at least two separate rounds of injections. Electron microscopy was performed as previously published (Ebarasi et al., 2009).

## Results

A female patient experienced a first episode of palpitation at 44 years of age and 5 years later noticed a weakness of the limb girdle muscle and inability to rise from a chair without the help of her hands. At the age of 53, she was diagnosed with sick sinus syndrome, sinoatrial block II, incessant focal atrial tachycardia, anterior left bundle branch block, and hemodynamically tolerated, sustained polymorphic ventricular tachycardia. An elevated serum CK level (863 U/L, normal range 29–200 U/L), as well as LDH (305 U/L, normal range 125–243 U/L) and myoglobin (233 ng/ml, normal range 0.0–106.0 ng/ml) was noticed. Biochemical level of Troponin I was within the normal range (0.37 ng/ml with reference interval 0–400 ng/ml), while CK-M level was slightly elevated (36.6 U/L with reference interval 0–24 U/L). Neurological examination confirmed reduced muscle strength, which in combination with morphological examination of muscle tissue resulted in the diagnosis of limb-girdle muscular dystrophy. Biochemical tests for anti-muscle antibodies and polymyositis-associated antibodies were negative. The cranial muscles, mimic, chewing, bulbar, and respiratory muscles were intact; muscle weakness was detected in the pelvic, proximal lower limb and upper girdle, and spine muscles. No muscle contracture, myotonia, or ataxia was observed; innervation of pelvic organs was intact and respiratory function assessment demonstrated intact respiratory muscles. Electromyography confirmed myopathic pattern and muscle CT of lower limbs detected symmetrical muscle wasting and decrease of muscle density due to fat substitution most prominent in the anterior and posterior hip muscles and gluteal muscles. Echocardiography demonstrated both left and right chambers enlargement, myocardial hypertrophy, hypokinetic interventricular septum, and basal segments of left ventricular posterior wall with preserved ejection fraction (55%). Notably, right ventricular and right atrial dimensions were increased in combination with thickening of the right ventricular myocardial wall (**Supplementary Material, Table S3**). Cardiac MRI demonstrated late gadolinium enchantment in the left ventricle, enlarged atria (left atrium dimension 52×39 mm and right atrium dimension 58×64 mm), and moderately enlarged left ventricle (end-diastolic dimension—46 mm) with moderate decrease in contractility. No signs of ischemic heart disease, hypertension, or chronic lung disease were registered. The family history reported no cardiac nor skeletal muscle disorders, but parental DNA was not available for genetic analysis and the relatives were not available for a detailed clinical investigation and phenotyping.

**Figure 1:**
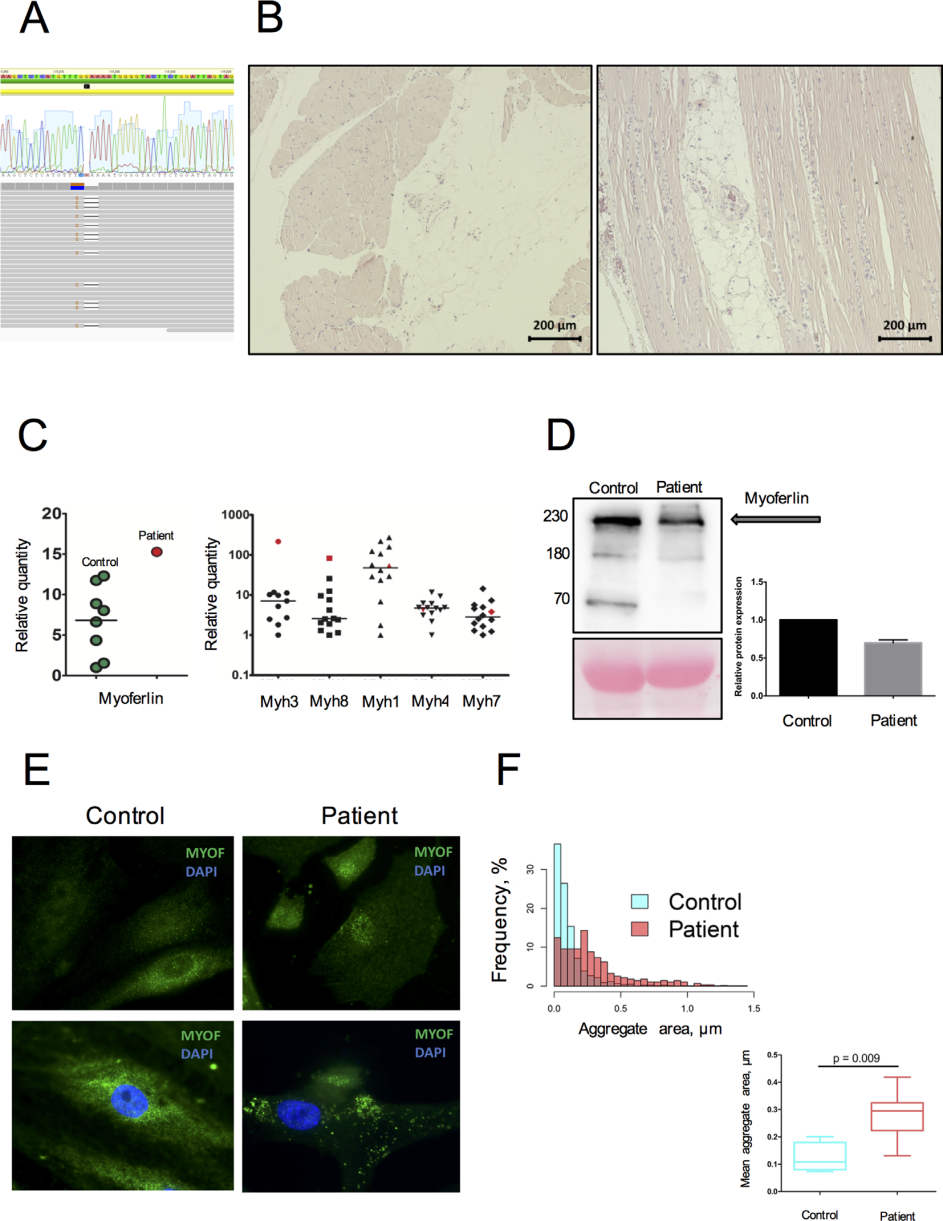
Genetic and morphological data on MYOF loss of function variant. **(A)** A heterozygous variant p.G859QfsTer8 resulted from cis-change of 2-neighbor mutated nucleotides (aligned RAW-reads and allele cloning of PCR products). **(B)** Morphological examination performed on *m. deltoideus*, following hematoxylin-eosin staining. Variation in fiber size, nuclear centralization, and fat accumulation along with inflammatory cells infiltration are often observed. **(C)** Increased mRNA expression of MYOF, embryonic (MYH3), and developmental (MYH8) myosins in the patient’s skeletal muscle tissue compared to donor samples (n = 13). The patient’s sample is represented in red; donor samples in green and black. **(D)** Decrease by 65% of the MYOF level in the patient’s SM-MPC when compared with healthy control. The detected fragments correspond to full size MYOF transcript (234 kDa) and suspected cleavage products (74 and 160 kDa) [30]. **(E)** Immunostaining of SM-MPC: low intensity of MYOF staining in the patient’s cells, patchy aggregate distribution, and nuclear intensification of the signal compared to control cells (×100). Histogram demonstrates the distribution of larger aggregates in the patient’s cells. **(F)** Quantitative characterization of MYOF aggregates. Representative frequency distribution of aggregate area in the control and patient SM-MPC and comparison of the mean aggregate area values for the control and the patient. The graph is presented as Tukey box-plot.

Primary genetic screening using a targeted panel of 108 cardiomyopathy-associated genes, including those associated with neuromuscular disorders, did not identify any likely disease-causing variants. Subsequently, WES was performed and after filtering, a list of non-synonymous and potentially affecting protein function variants was further analyzed (**Supplementary Material, Table S4**). Data processing, variant filtering, and assessment are described in **Supplementary Material (Figure S1**, Filtering Strategy). This analysis identified two neighboring cis variants in *MYOF* (MYOF chr10:95066186-95242074, GRCh37.p13) resulting in a frameshift mutation (NM_133337:c.[2576delG; 2575G>C], p.G859QfsTer8) (**Figure 1A**). The variant is classified as likely pathogenic according to the 2018 American College of Medical Genetics and Genomics (ACMG) guidelines (criteria codes PS3, PM2, PM4).

Morphological examination of the patient’s skeletal muscle demonstrated fiber size variation, splitting, and nuclear centralization in more than 30% of fibers in combination with fat infiltration and fibrosis (**Figure 1B**). Signs of muscle degeneration/regeneration and fatty infiltration were observed. An upregulation of *MYOF* mRNA expression in the patient’s skeletal muscle tissue was detected compared to healthy donors. This was accompanied by increased expression in skeletal muscle biopsy of embryonic and developmental myosins *MYH3* and *MYH8*, while expression of adult myosin forms (*MYH1*, *MYH4*, *MYH7*) was within the normal range (**Figure 1C**). In contrast, Western blot analysis performed on the patient’s SM-MPC confirmed a decreased expression (65%) of *MYOF* in the patient’s SM-MPC with almost complete absence of the low molecular weight isoform (**Figure 1D**). Staining of the patient’s SM-MPC with anti-MYOF antibody further confirmed a decreased staining intensity and formation of intracellular aggregates of larger diameter and greater number compared to control cells (**Figure 1E, F**). Notably, in a primary culture of the patient’s SM-MPC, MYOF was often localized in the nuclear area, which was not observed in control cells.

**Figure 2:**
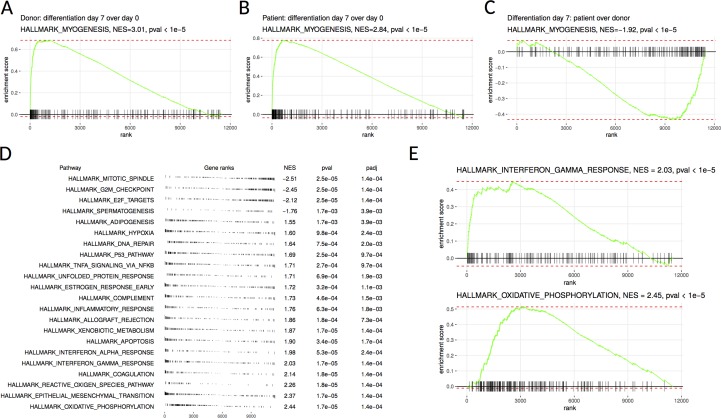
Gene set enrichment analysis of donor-derived and patient-derived SM-MPC. **(A, B)** Differentiated cells are highly enriched in hallmark myogenesis pathway both in the donor **(A)** and the patient’s **(B)** cells. **(C)** Myogenesis differentiation is decreased in the patient’s cells compared to donor cells. **(D)** Pathways from MSigDB hallmark database showing significant enrichment in donor versus patient comparison. Pathways are ordered according to normalized enrichment score (NES). Positive NES values correspond to pathways upregulated in the patient’s cells and negative values correspond to downregulated pathways. **(E)** Individual enrichment plots for interferon gamma response and oxidative phosphorylation pathways.

RNA sequencing was performed on the patient’s SM-MPC and differentiated myotubes in order to analyze the effect of the *MYOF* which is likely pathogenic variant on intracellular pathways. The analysis revealed dysregulation of multiple pathways including inflammation and mitochondrial metabolism. Myogenesis was activated in both patient and control samples after differentiation, as seen by *MYH1*, *MYH2*, *MYL2*, *TNNC2*, and *CASQ2* expression levels (**Figure 2A, B**). Consistently with the Western blot analysis, following RNA sequencing, MYOF mRNA showed twofold down-regulation in patient undifferentiated cells compared to donor cells [56.1 transcripts per million (TPM) compared to 112.25 TPM, **Supplementary Material, Table S5**]. However, a comparison of the patient’s samples with the corresponding control samples after differentiation showed a down-regulation of the myogenesis pathway genes in the patient’s samples (**Figure 2C**). This was accompanied by activation of inflammatory and hypoxia-induced genes (**Figure 2D**). Additionally, mitochondrial metabolism pathways (e.g., *SLC25A4*, *ATP5D*, *NQO2*, *NDUFB7*) and several inflammation pathways including IFNγ response (e.g., *CFB*, *IL6*, *IFI30*, *ICAM1*, *IFI27*, *CCL2*) were up-regulated in the patient’s SM-MPC (**Figure 2E**).

**Figure 3:**
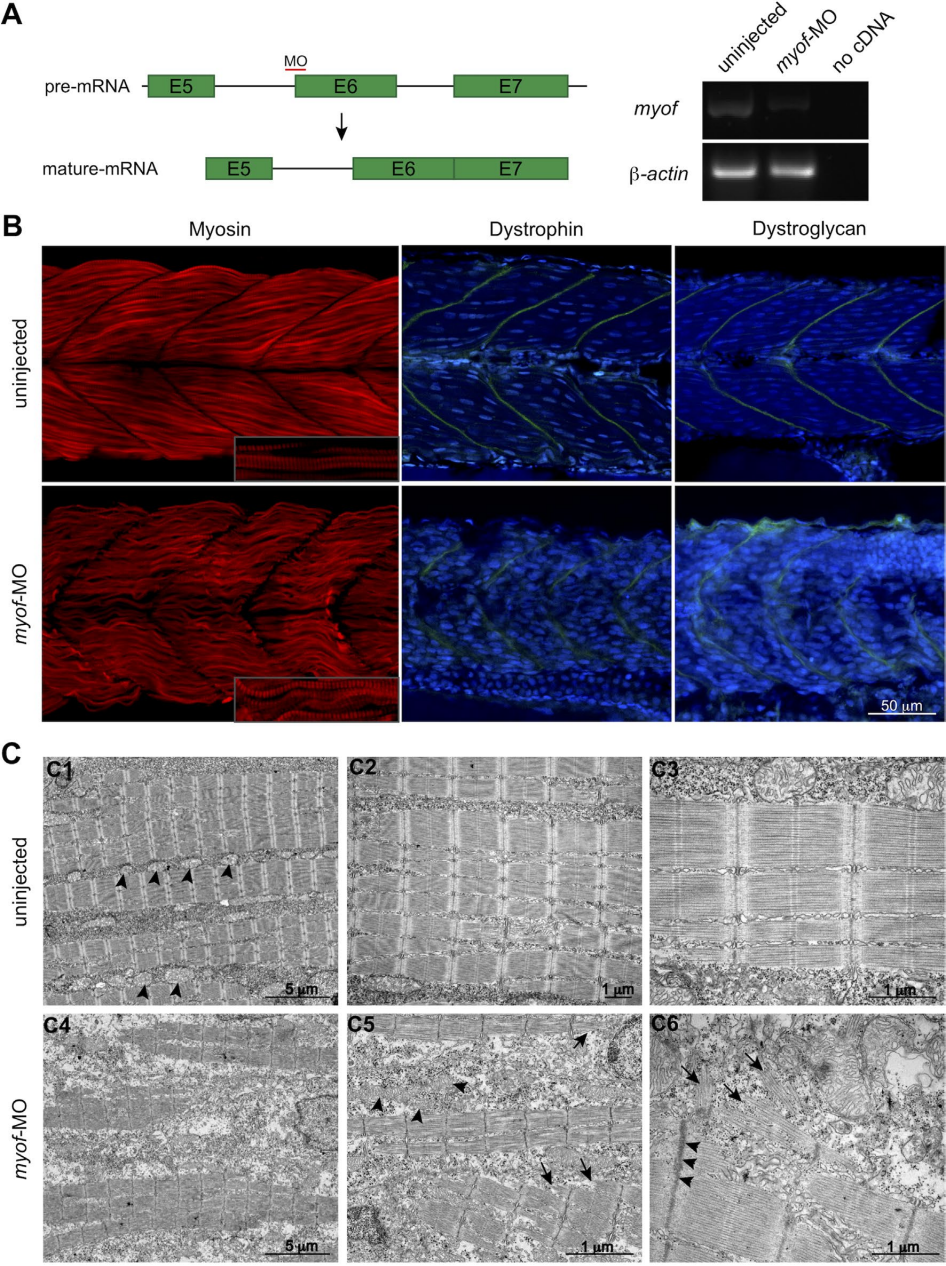
Loss of Myof affects the skeletal muscle of zebrafish embryos. **(A)** *myof* knockdown was achieved by injecting a splice-target morpholino (*myof*-MO). **(B)** Loss of Myof results in wavy and abnormally patterned myofibers (Myosin, red), and disruption of the myosepta, seen by a decreased intensity in the staining and increased width (Dystrophin and Dystroglycan, green). **(C)** Analysis of the myofiber ultrastructure by electron microscopy shows disruption of myofiber structure (C4) and their sarcomeres (arrows, C5, C6), abnormally shaped and distributed mitochondria (arrowheads, C5), when compared to control mitochondria (arrowheads, C1), and Z-disc thickening (arrowheads, C6).

To further elucidate the role of MYOF in myogenesis, we performed knockdown experiments in the zebrafish embryo using a splice-target morpholino (*myof*-MO). This MO binds to the intron 5–exon 6 boundary and is predicted to block the splicing of the intron (**Figure 3A**) and target the mRNA for degradation by non-sense mediated decay (Lykke-Andersen and Jensen, 2015). RT-PCR analysis of *myof*-MO injected embryos showed a decrease in the WT *myof* mRNA when compared with uninjected siblings (**Figure 3A**), suggesting retention of intron 5. Loss of Myof in zebrafish resulted in a myopathy-like phenotype, including wavy fibers and disrupted Myosin patterning (in red, **Figure 3B**). Other members of the Ferlin family of proteins are localized at the Z-disc and myosepta, with knockdown of *dysferlin* reported to result in myosepta disruption (Kawahara et al., 2011). Since the myosepta of *myof*-MO injected embryos appeared to be wider than in control siblings, we immunolabelled for proteins of the myosepta, Dystrophin and Dystroglycan, and found a wider and less dense distribution of these proteins, suggesting myosepta disruption (in green, **Figure 3B**). Analysis of the myofibers using electron microscopy showed a general disruption of the myofibrils (**Figure 3C4; arrows, C5 and C6**), with abnormally organized and misshaped mitochondria (arrowheads, **Figure 3C5**), Z-disc streaming (arrowheads, **Figure 3C6**), and widening of triads.

## Discussion

Here we have presented a clinical case of cardiomyopathy associated with limb-girdle type muscular dystrophy where WES strategy allowed the identification of a truncating variant in myoferlin (*MYOF*), c.[2576delG; 2575G>C], p.G859QfsTer8. The clinical phenotype in combination with *in vitro* and *in vivo* studies allowed us to classify the detected MYOF variant as likely pathogenic and to link for the first time *MYOF* with a human genetic disease.

*MYOF* is mainly expressed in heart and skeletal muscle where, similar to *DYSF*, it participates in membrane repair and muscle regeneration after injury through the activation of the NFAT-dependent promoter (Demonbreun et al., 2010a; Han, 2011). It is also involved in myoblast and satellite cell fusion into mature myofibers during embryo development and postnatally (Doherty et al., 2005). Moreover, MYOF modulates inflammatory and growth response by participation in IGF1R, EGFR, and VEGFR2 trafficking and recycling (Demonbreun et al., 2010b; Turtoi et al., 2013). The recent implication in exosome maturation made it an important protein for cell–cell communication both in myogenesis and tumorigenesis (Forterre et al., 2014; Blomme et al., 2016). In spite of their structural similarity and tissue-specific expression, MYOF and DYSF do not present with overlapping functions. In fact, they have distinct time-dependent expression profiles as well as slightly distinct subcellular localization and Ca^2+^-dependent cleavage sites (Piper et al., 2017). In contrast to DYSF, Ca^2+^-dependent MYOF cleavage occurs in resting cells leading to constant basal release of MYOF cleavage products (Redpath et al., 2014; Piper et al., 2017). The absence of overlapping function between MYOF and DYSF is further supported by the fact that MYOF can partly compensate for DYSF loss *in vitro*, while having a limited ability to compensate DYSF loss *in vivo* (Inoue et al., 2006; Lostal et al., 2012).

The described clinical phenotype in combination with morphological data and CK elevation allowed us to suggest *MYOF* as a strong candidate for being a disease-causing gene. A combination of *in vitro* and *in vivo* studies allowed us to classify the identified variant as likely pathogenic and for the first time link *MYOF* to human inherited disease. Of note, we observed a much stronger MYOF staining in the nucleus of patient-derived muscle stromal cells compared to control cells—a phenomenon previously reported by Davis et al. (2000). This, in part, may be attributed to the role of MYOF in chaperoning phosphorylated STAT3 into the nucleus under IL-6 induced signaling (Yadav et al., 2017), and supported by the activation of the IFNγ-proinflammatory pathway and IL-6 signaling in *MYOF*-mutant SM-MPC during differentiation. Together, the RNA signature of proinflammatory pathway activation and downregulation of myogenesis in *MYOF*-mutant cells confirms the role of *MYOF* in myogenesis and modulation of inflammatory signaling. The inflammation expression pattern was confirmed by comparison to GSE26852 dataset (gene expression analysis of facioscapulohumeral muscular dystrophy muscle with different MRI pattern) (Tasca et al., 2012). There, genes upregulated in T2-short tau inversion recovery positive samples corresponding to more inflammation muscle phenotype (T2-STIR+) significantly overlap with genes upregulated in the patient’s samples (p-value of GSEA test < 1e−5). The increase in embryonic and developmental regeneration markers such as *MYH3* and *MYH8* in the patient’s muscle tissue supports the ongoing inflammation-regeneration process. This, in part, can explain the activation of *MYOF* expression in the adult patient’s tissue—a phenomenon not observed in healthy donor skeletal muscle due to very low *MYOF* expression in adult matured myofibers (Schiaffino et al., 2015). However, on a single cell level *in vitro* *MYOF* expression in the patient’s SM-MPC was decreased compared to control cells suggesting that the p.G859QfsTer8 variant causes loss of *MYOF* function.

Loss of Myof in zebrafish results not only in a myopathy-like phenotype and myosepta disruption, but also abnormal sarcomeric organization, disrupted myosin patterning, mitochondria abnormalities, and Z-disc streaming. Other members of the Ferlin family are localized at the Z-disc and myosepta, with knockdown of *dysferlin *reported to result in myosepta disruption (Kawahara et al., 2011). Given the lack of a working antibody against Myof in zebrafish, we were not able to verify the localization in the zebrafish skeletal muscle. Nevertheless, we suggest that Myof may present the same distribution as its family members, supported by the resulting phenotype when *myof* is knocked down. This is the first report on ultrastructural sarcomeric and Z-line abnormalities induced by *MYOF* loss, however, well in line with the previous observations on the role of *MYOF* in T-tubular system organization and remodeling (Demonbreun et al., 2014). Disorganization of T-tubular system and triads can, in part, contribute to proarrhythmic phenotypes observed in the patient described here. Previous reports have showed a conservation of the DysF domains in the Ferlin family, and their importance for normal protein function and implication in disease (Therrien et al., 2006; Patel et al., 2008). We therefore suggest that both loss and truncation of MYOF may result in similar phenotypes, which is shown here in the *in vitro* experiments. It is, however, not entirely clear whether disease-causing variants in *MYOF* act as gain-of-function or haploinsufficient variants.

A disease-causative role of the identified *MYOF* loss of function variant is supported by analyses of the patient’s satellite cells, RNA sequencing data, morphological and immunohistochemical studies, as well as recapitulation of the muscle phenotype in zebrafish experiments. The clinical presentation resembles that observed when *dysferlin* is knocked down (Kawahara et al., 2011). In aggregate, our study provides the first evidence of *MYOF* gene as being associated with human disease phenotype and broadens the field for further research of the Ferlin protein family in connection to disorders of cardiac and skeletal muscle. The identification of singe allele damage leading to approximately 40% reduction in RNA and protein level in association with a late-onset clinical phenotype suggests an autosomal dominant inheritance pattern. However, as for many other neuromuscular disorder-associated genes, such as *LMNA*, *DYSF*, *TTN*, *MYH7*, and *COL6A2*, both dominant and recessive mechanisms are possible, depending on the number of alleles damaged and the effects on protein levels (Bonne et al., 2017). Analysis of parental DNA and detailed phenotyping of close relatives would shed more light on the specific mechanism involved in the patient reported here, and the unavailability of these data represents a limitation of the current study.

Our data indicate that the identified *MYOF* variant acts as a loss of function allele and, thus, can potentially lead to deleterious functional effects due to decreased RNA and protein levels. However, more than 70 loss of function *MYOF* variants have been reported in public databases such as ExAC and gnomAD. Some of these affect alternative non-coding transcripts and no phenotype information is provided. Notably, only about 30% of the individuals in those datasets are expected to be older than the patient presented here. Even so, incomplete or low penetrance of MYOF associated muscle disease remains a possibility, and further studies are necessary to determine the functional and clinical effects of loss of *MYOF*.

Of note, we observed a stronger MYOF staining in the nucleus of the patient-derived SM-MPC to control cells previously reported by Davis et al. (2000). This may, in part, be attributed to the role of MYOF in chaperoning phosphorylated STAT3 into the nucleus under IL-6 induced signaling (Yadav et al., 2017), which was supported even further by our finding of activation of the IFNγ-proinflammatory pathway and IL-6 signaling in *MYOF*-mutant cells during differentiation. Together, the RNA signature of proinflammatory pathway activation and downregulation of myogenesis in *MYOF*-mutant cells are in line with previously proposed roles of *MYOF* in myogenesis and modulation of inflammation signaling (Demonbreun et al., 2010; Turtoi et al., 2013; Forterre et al., 2014).

## Conclusion

In summary, we present a first report of a loss of function *MYOF* variant in a patient with cardiomyopathy and limb-girdle type muscular dystrophy phenotype. The disease-causative role of the identified variant is supported by morphological and molecular studies, RNA sequencing data, as well as recapitulation of muscle phenotype in a zebrafish model. Our study provides the first evidence of the *MYOF* gene being associated with a human disease phenotype and broadens the field for further research on Ferlin protein family in connection with disorders of cardiac and skeletal muscles.

## Ethics Statement

The study was performed according to the Declaration of Helsinki, and approval was obtained from the Ethical Review Boards of Karolinska Institute and Almazov National Medical Research Centre, approval number 2016/54. Written informed consent was obtained from both the study subject and healthy donors prior to the investigation, including a consent for publication. All procedures with zebrafish were performed in accordance with standard operating procedures approved by the Stockholm Ethical Board for Animal Experiments (permit number 13063-2017).

## Author Contributions

AKi, RV, and AKn contributed to the conception and design of the study, analysis, and interpretation of the data and drafting of the manuscript. TS and AL made contributions to the conception and design of the study and revision of the manuscript critically. AKo contributed to the study concept and research design and wrote the manuscript. GS, DR, LM, and EM took part in the analysis and interpretation of the data and have been involved in revising the manuscript critically. AS, AP, and JJ performed bioinformatics analysis. AKh, RV, YF, RD, KS, and NS conducted the experiments and performed the analysis and interpretation of the data. All authors have read and approved the final version of the manuscript.

## Funding

This work was supported by Russian Scientific Foundation (14-15-00745-П) for study design, data collection and analysis, sequencing and morphological studies, cell culturing, and expression analysis; Swedish Society for Medical Research and Swedish Research Council (2013-2603; 2017-02936), ALF funding (20140240, 20170831), Stiftelsen Frimurare Foundation, Promobilia for zebrafish model design and analysis and confocal imaging, Government of Russian Federation (08-08) for bioinformatics and statistical analysis.

## Conflict of Interest Statement

The authors declare that the research was conducted in the absence of any commercial or financial relationships that could be construed as a potential conflict of interest.

## Abbreviations

LGMD2B, limb-girdle muscle dystrophy 2B; WES, whole exome sequencing; FCS, fetal calf serum; DMEM, Dulbecco medium Eagle’s modified; MRI, magnetic resonance investigation; CK, creatine kinase; LDH, lactate dehydrogenase; SM-MPC, skeletal muscle mesenchymal progenitor cells; MO, morpholino; TPM, transcripts per million; T2-STIR+, T2-short tau inversion recovery positive samples; ExAC, The Exome Aggregation Consortium; gnomAD, The Genome Aggregation Database.
