# Supplementary material for: Truncating Variant in Myof Gene Is Associated With Limb-Girdle Type Muscular Dystrophy and Cardiomyopathy
Source: Front Genet. 2019 Jun 26;10:608. doi: 10.3389/fgene.2019.00608 (PMC6607695; doi:10.3389/fgene.2019.00608)
Supplement: Supplementary file 1 [file Table_1.docx]

**Supplementary material**

**Figure S1. Filtering strategy for the variants, obtained after WES.**

**Figure S2. Characterisation of SMSC immunophenotyped and differentiation potential.**


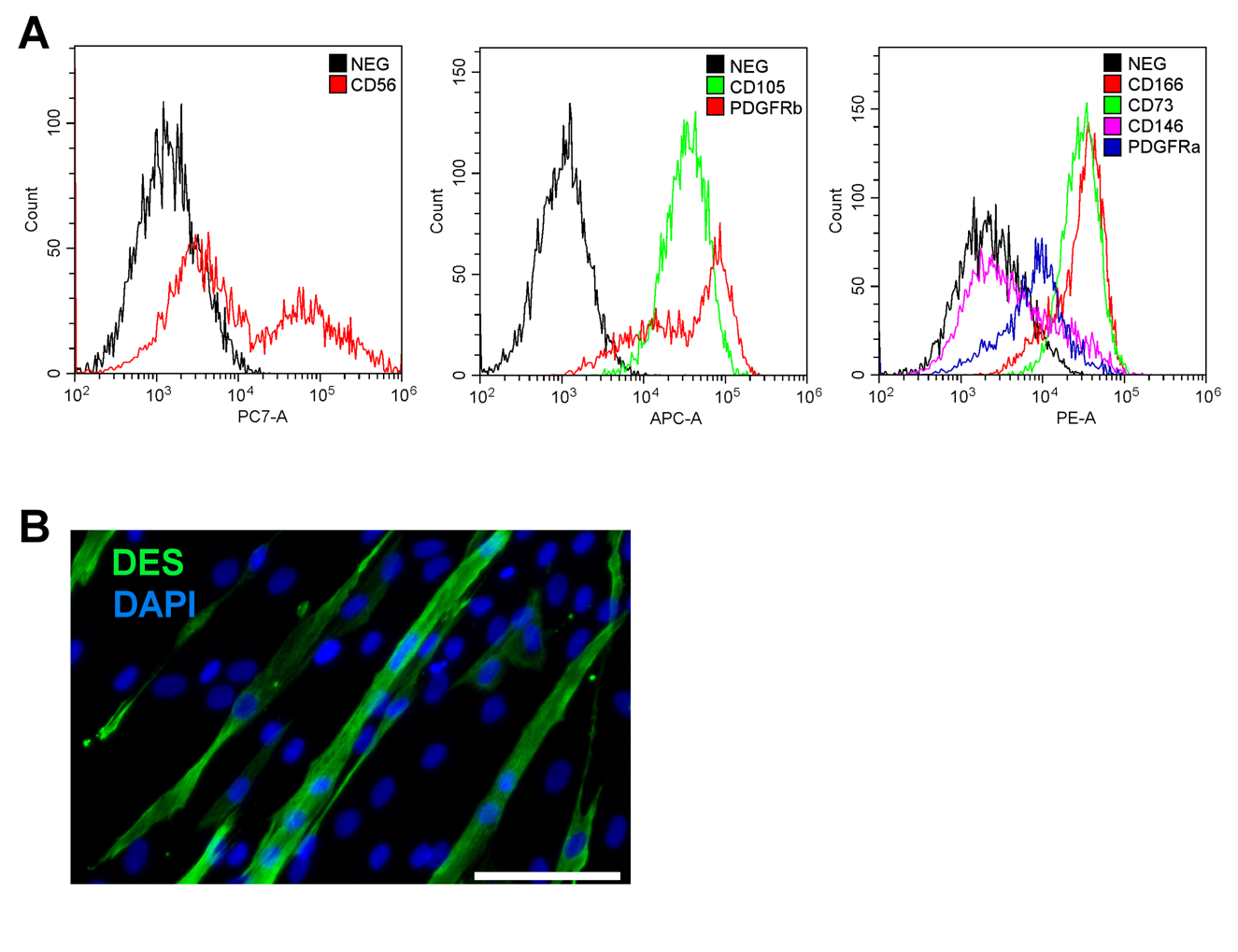


A. Representative flow cytometric histograms demonstrate immunophenotype of skeletal muscle stromal cells isolated from muscle biopsy. Virtually all SMSC cells were positive for mesenchymal markers CD73, CD105 and CD166, but not for CD146; the substantial fraction of SMSC samples was positive for PDGFRa/PDGFRb, and about 30-40% were positive for NCAM/CD56, a reliable molecular marker of satellite cells and myoblasts in human skeletal muscle. B. Myotubes after 7 days of myogenic differentiation. Desmin staining (green). Nuclei are labelled with DAPI (blue). Scale bars represent 100μ.

**Table S1. List of studied genes.**

(Almazov_CardioMyoPathy_Arrhythmia_Noonan. Agilent Design ID: 27291-1393420132)

| **TargetID** | Interval | Cover. | High  Cover. | Low  Cover. |
| --- | --- | --- | --- | --- |
| **ABCC9** | chr12:21953968-22089618 | 100.00 | 41 | 0 |
| **ACTC1** | chr15:35082603-35087019 | 100.00 | 6 | 0 |
| **ACTN2** | chr1:236849964-236925929 | 99.82 | 23 | 0 |
| **AKAP9** | chr7:91570404-91739483 | 99.93 | 52 | 0 |
| **ANK2** | chr4:113825640-114309894 | 100.00 | 58 | 0 |
| **ANKRD1** | chr10:92672613-92680794 | 100.00 | 9 | 0 |
| **BAG3** | chr10:121411178-121436804 | 100.00 | 5 | 0 |
| **BRAF** | chr7:140426284-140624513 | 100.00 | 21 | 0 |
| **CACNA1C** | chr12:2162719-2800375 | 99.95 | 53 | 0 |
| **CACNA2D1** | chr7:81579698-82072785 | 100.00 | 42 | 0 |
| **CACNB2** | chr10:18429656-18828663 | 99.78 | 20 | 0 |
| **CALM1** | chr14:90863565-90871071 | 100.00 | 7 | 0 |
| **CALR3** | chr19:16589932-16606950 | 100.00 | 9 | 0 |
| **CASQ2** | chr1:116243852-116311172 | 100.00 | 11 | 0 |
| **CAV3** | chr3:8775553-8787563 | 100.00 | 2 | 0 |
| **CBL** | chr11:119077118-119170501 | 100.00 | 16 | 0 |
| **CRYAB** | chr11:111779478-111782458 | 100.00 | 3 | 0 |
| **CSRP3** | chr11:19204207-19214005 | 99.85 | 5 | 0 |
| **DES** | chr2:220283175-220290722 | 100.00 | 9 | 0 |
| **DMD** | chrX:31132798-33357392 | 99.92 | 88 | 0 |
| **DMPK** | chr19:46273729-46285639 | 100.00 | 16 | 0 |
| **DSC2** | chr18:28647971-28681944 | 100.00 | 18 | 0 |
| **DSG2** | chr18:29078205-29126716 | 100.00 | 15 | 0 |
| **DSP** | chr6:7542139-7586121 | 100.00 | 25 | 0 |
| **DTNA** | chr18:32335931-32470414 | 100.00 | 27 | 0 |
| **EMD** | chrX:153607835-153609567 | 100.00 | 6 | 0 |
| **EYA4** | chr6:133595909-133852374 | 100.00 | 21 | 0 |
| **FHL1** | chrX:135251952-135292194 | 100.00 | 10 | 0 |
| **FHL2** | chr2:105977730-106015563 | 100.00 | 8 | 0 |
| **FKTN** | chr9:108337304-108402414 | 99.68 | 12 | 0 |
| **FXN** | chr9:71650689-71714860 | 100.00 | 6 | 0 |
| **GAA** | chr17:78078376-78093140 | 100.00 | 19 | 0 |
| **GLA** | chrX:100652787-100662901 | 100.00 | 7 | 0 |
| **GPD1L** | chr3:32148194-32207412 | 100.00 | 8 | 0 |
| **HCN4** | chr15:73614812-73660621 | 100.00 | 8 | 0 |
| **HRAS** | chr11:532626-534332 | 100.00 | 5 | 0 |
| **ILK** | chr11:6625492-6631852 | 100.00 | 12 | 0 |
| **JPH2** | chr20:42743426-42815355 | 100.00 | 6 | 0 |
| **JUP** | chr17:39775836-39928116 | 100.00 | 20 | 0 |
| **KCND3** | chr1:112318689-112525358 | 100.00 | 7 | 0 |
| **KCNE1** | chr21:35821533-35821942 | 100.00 | 1 | 0 |
| **KCNE1L** | chrX:108867811-108868259 | 100.00 | 1 | 0 |
| **KCNE2** | chr21:35742768-35743159 | 100.00 | 1 | 0 |
| **KCNE3** | chr11:74168287-74168618 | 100.00 | 1 | 0 |
| **KCNH2** | chr7:150642443-150675011 | 100.00 | 16 | 0 |
| **KCNJ2** | chr17:68171171-68172474 | 100.00 | 1 | 0 |
| **KCNJ5** | chr11:128781159-128786636 | 100.00 | 2 | 0 |
| **KCNJ8** | chr12:21918647-21926560 | 100.00 | 2 | 0 |
| **KCNQ1** | chr11:2466319-2869243 | 100.00 | 18 | 0 |
| **KRAS** | chr12:25362719-25398328 | 100.00 | 5 | 0 |
| **LAMA4** | chr6:112430630-112575362 | 100.00 | 44 | 0 |
| **LAMP2** | chrX:119562329-119603034 | 100.00 | 11 | 0 |
| **LDB3** | chr10:88428439-88492743 | 100.00 | 16 | 0 |
| **LMNA** | chr1:156084700-156109640 | 99.71 | 16 | 1 |
| **MAP2K1** | chr15:66679676-66782963 | 100.00 | 11 | 0 |
| **MAP2K2** | chr19:4090586-4123882 | 100.00 | 11 | 0 |
| **MRPL3** | chr3:131181557-131221837 | 100.00 | 12 | 0 |
| **MYBPC3** | chr11:47353412-47374208 | 100.00 | 34 | 0 |
| **MYH6** | chr14:23851239-23876442 | 99.44 | 37 | 0 |
| **MYH7** | chr14:23882053-23902951 | 99.55 | 38 | 0 |
| **MYL2** | chr12:111348871-111358343 | 88.61 | 6 | 1 |
| **MYL3** | chr3:46899724-46904890 | 100.00 | 6 | 0 |
| **MYLK2** | chr20:30407374-30421610 | 100.00 | 12 | 0 |
| **MYOM1** | chr18:3067250-3215231 | 100.00 | 38 | 0 |
| **MYOZ2** | chr4:120057671-120107365 | 100.00 | 5 | 0 |
| **MYPN** | chr10:69866472-69970222 | 100.00 | 22 | 0 |
| **NEBL** | chr10:21074666-21462772 | 100.00 | 34 | 0 |
| **NEXN** | chr1:78381782-78408608 | 100.00 | 14 | 0 |
| **NF1** | chr17:29422318-29705959 | 99.63 | 59 | 1 |
| **NOS1AP** | chr1:162039958-162353331 | 100.00 | 13 | 0 |
| **NRAS** | chr1:115251146-115258791 | 100.00 | 4 | 0 |
| **PDLIM3** | chr4:186423438-186456598 | 99.45 | 9 | 0 |
| **PKP2** | chr12:32945348-33049675 | 100.00 | 14 | 0 |
| **PLN** | chr6:118880075-118880253 | 100.00 | 1 | 0 |
| **PRKAG2** | chr7:151254277-151573715 | 99.87 | 19 | 0 |
| **PSEN1** | chr14:73614718-73686007 | 100.00 | 11 | 0 |
| **PSEN2** | chr1:227068337-227083290 | 100.00 | 11 | 0 |
| **PTPN11** | chr12:112856906-112942578 | 100.00 | 16 | 0 |
| **RAF1** | chr3:12626003-12660230 | 99.57 | 17 | 0 |
| **RANGRF** | chr17:8192097-8193264 | 100.00 | 4 | 0 |
| **RBM20** | chr10:112404203-112595746 | 100.00 | 15 | 0 |
| **RYR2** | chr1:237205812-237995957 | 99.70 | 110 | 1 |
| **SCN1B** | chr19:35521715-35530615 | 99.64 | 5 | 0 |
| **SCN3B** | chr11:123504841-123524519 | 100.00 | 5 | 0 |
| **SCN4B** | chr11:118007732-118023398 | 100.00 | 5 | 0 |
| **SCN5A** | chr3:38591802-38674808 | 99.73 | 28 | 0 |
| **SCO2** | chr22:50962030-50962850 | 100.00 | 1 | 0 |
| **SDHA** | chr5:218461-256545 | 97.59 | 15 | 1 |
| **SGCD** | chr5:155756577-156186411 | 100.00 | 9 | 0 |
| **SHOC2** | chr10:112724107-112771586 | 100.00 | 8 | 0 |
| **SLC25A3** | chr12:98987747-98995316 | 100.00 | 8 | 0 |
| **SLMAP** | chr3:57743369-57913125 | 100.00 | 24 | 0 |
| **SNTA1** | chr20:31996303-32031436 | 100.00 | 8 | 0 |
| **SOS1** | chr2:39212955-39347573 | 99.65 | 23 | 1 |
| **SPRED1** | chr15:38545377-38643875 | 100.00 | 7 | 0 |
| **TAZ** | chrX:153640171-153649353 | 100.00 | 10 | 0 |
| **TCAP** | chr17:37821603-37822372 | 100.00 | 2 | 0 |
| **TGFB3** | chr14:76425520-76447246 | 100.00 | 7 | 0 |
| **TMEM43** | chr3:14166684-14183305 | 100.00 | 13 | 0 |
| **TMPO** | chr12:98909537-98941646 | 100.00 | 10 | 0 |
| **TNNC1** | chr3:52485281-52488041 | 100.00 | 6 | 0 |
| **TNNI3** | chr19:55663192-55668967 | 100.00 | 8 | 0 |
| **TNNT2** | chr1:201328328-201342392 | 100.00 | 21 | 0 |
| **TPM1** | chr15:63335019-63363381 | 100.00 | 16 | 0 |
| **TRDN** | chr6:123539736-123957930 | 98.85 | 48 | 1 |
| **TRPM4** | chr19:49661114-49714765 | 100.00 | 25 | 0 |
| **TTN** | chr2:179391729-179682294 | 98.80 | 346 | 18 |
| **VCL** | chr10:75757956-75877977 | 100.00 | 22 | 0 |

**Table S2. List of primers used in Q-PCR experiments.**

| Gene name | Forward primer | Reverse pimer |
| --- | --- | --- |
| hMYOF | TTCTCATCTTCGGGAACTGGG | ATCCCGTGTACTCTCTGGGG |
| hMYH3 | GTGGTGGACTCAAAGGAAGAA | GGTCCTGTTGTCCTCAGTTT |
| hMYH8 | TTTCCACCAAGAACCCAGAG | CACTCATGGCTGCGATTTATTT |
| hMYH1 | CACCACCAACCCATACGATTA | GGCACTATCTGTAGCCAA |
| hMYH4 | CTCCATCTCTGACAATGCCTATC | TTCGTGTTCACAGTCTTCCC |
| hMYH7 | GACCTCAAGAAGGATGTCTTCG | GGTCACTGTCTTGCCATACTC |
| hGAPDH | AATGAAGGGGTCATTGATGG | AAGGTGAAGGTCGGAGTCAA |

##### Table S3. Echocardiographic data of proband.

| Parameter | dimensions |
| --- | --- |
| LVESD (mm) | 29 |
| LVEDD (mm) | 59 |
| LVEDV (ml) | 123 |
| IVSd (mm) | 10 |
| LVPWd (mm) | 11 |
| LAD (mm) | 40 |
| RAD (mm) | 53 |
| RVEDD (mm) | 28 |
| RVAWd (mm) | 4.5 |
| TAPSE (mm) | 15 |
| PAPs (mmHg) | 25 |
| MI (g/M^2^) | 145 |
| EF/FS | 55/44 |

**EF** – ejection fraction, **FS** – fractional shortening, **IVSd** – interventricular septum measured in end diastole, **LAD** – left atrium dimension, **LV mass** – left ventricular mass,  **LVEDD** - left ventricular end diastolic dimension, **LVEDV** - left ventricular end diastolic volume, **LVESD** - left ventricular end systolic dimension, **LVPWd** – left ventricular posterior wall thickness in end diastole, **TAPSE** – tricuspid annular plane systolic excursion, **PAPs** – pulmonary artery pressure, **RAD** – right atrium dimension, **RVEDD** - right ventricular end diastolic dimension, **RVAWd** – right ventricular anterior wall thickness in end diastole, **MI**-myocardial mass index.
